# Evolution of vascular normalization strategies: from anti-angiogenesis to remodeling the tumor microenvironment

**DOI:** 10.3389/fimmu.2026.1959437

**Published:** 2026-10-30

**Authors:** Qian Huang, Yujing Guo, Sha Yin, Lunbiao Gan, Zehua Liu, Wenxiu Ru, Yu Lu, Sijia Di, Fengze Nie, Siyu Yao, Huanhuan Wan, Le Liu, Jiaqi Ning, Fa Yang, Weijun Qin, Weihong Wen

**Affiliations:** 1 School of Life Sciences and Technology, Northwestern Polytechnical University, Xi’an, China; 2 Department of Urology, Xijing Hospital, Fourth Military Medical University, Xi’an, China

**Keywords:** angiogenesis, immune reprogramming, multicellular interactions, tumor microenvironment, vascular normalization

## Abstract

Although anti-angiogenic therapy has been widely used in the treatment of solid tumors, angiogenesis inhibition alone frequently induces acquired resistance and exacerbates tumor hypoxia, paradoxically promoting tumor progression. The concept of tumor vascular normalization has offered a new perspective to address this dilemma, shifting the therapeutic strategy from “vessel destruction” to “vessel repair.” This review systematically elucidates the pathological characteristics of aberrant tumor vasculature and the cascading responses driven by these abnormalities. We summarize the molecular regulatory networks underlying vascular normalization across multiple dimensions, including VEGF signaling, hypoxia-inducible factors, extracellular matrix remodeling, metabolic reprogramming, and immune cell coordination. On this basis, this review further explores how vascular normalization systematically reshapes the tumor immune microenvironment by alleviating immunosuppression, promoting T cell infiltration, supporting an IFN-γ-mediated positive feedback loop, and coordinating with tertiary lymphoid structure formation. Finally, we dissect the multicellular interaction networks among tumor cells, endothelial cells, pericytes, and immune cells, and propose future directions including the vascular-immune dual-biomarker system and the targeting of pericyte phenotypic switching. This review provides a systematic framework for understanding the synergistic mechanisms linking vascular normalization and immune reprogramming, and lays a foundation for optimizing combination therapeutic strategies.

## 1 Introduction

The rapid growth of solid tumors demands increased oxygen and nutrient supply. To meet this demand, tumors stimulate the sprouting of new blood vessels from pre-existing blood vessels, a process known as angiogenesis. Tumor growth and metastasis are highly dependent on new blood vessel formation (1). In recent years, anti-angiogenic therapies, including VEGF/VEGFR-targeted antibodies, receptor tyrosine kinase inhibitors, and agents directed against other angiogenic pathways, have demonstrated clinical efficacy in various solid tumors. However, angiogenesis inhibition alone frequently induces adaptive resistance and exacerbates hypoxia, paradoxically promoting tumor invasion and immune evasion (2, 3). Therefore, moving beyond mere “anti-angiogenesis” to explore more effective vascular regulatory strategies has become a critical issue in the field of oncology.

The concept of tumor vascular normalization has provided a new avenue for addressing this dilemma. First proposed by Rakesh K. Jain in 2001, this concept suggests that the judicious use of anti-angiogenic agents or other interventions can remodel aberrant vessels into structurally more mature and functionally better-perfused “normalized” vessels (4). Over the past 25 years, this concept has evolved from a debated hypothesis into an increasingly supported therapeutic strategy, backed by substantial preclinical and emerging clinical evidence that vascular normalization can improve drug delivery, enhance immune cell infiltration, and increase tumor sensitivity to chemotherapy, radiotherapy, and immunotherapy (5). This theory shifted the research focus from simple angiogenesis inhibition toward the restoration and optimization of vascular function, thereby opening new perspectives for combination therapy.

In recent years, advances in single cell sequencing and spatial transcriptomics have provided new tools for dissecting multicellular interactions. Vascular normalization enhances T cell infiltration, while activated CD8^+^ T cell-derived IFN-γ further promotes vascular normalization, forming a positive feedback loop (6). Endothelial cells not only serve as components of the vessel wall but also actively shape the tumor immune microenvironment by secreting chemokines and immunomodulatory factors that regulate lymphocyte recruitment, retention, and spatial organization (6). Notably, these analyses further reveal that tumor endothelial cells (TECs) are not a uniform population but comprise functionally distinct subsets with diverse immune-regulatory and metastasis-promoting properties, which may fundamentally influence the efficacy of vascular normalization strategies (1, 7). These findings highlight the existence of a complex multicellular interaction network among the vascular system, the immune system, and tumor cells within the tumor microenvironment. Systematically dissecting these interactions is essential for optimizing therapeutic strategies. However, most existing reviews have largely focused on individual molecular mechanisms of vascular normalization, with limited systematic integration of multicellular interaction networks. This review systematically delineates the pathological features of aberrant tumor vasculature, the regulatory mechanisms of vascular normalization, its role in remodeling the tumor immune microenvironment, and the relevant multicellular interaction networks, thereby providing a theoretical foundation for optimizing combination therapies.

## 2 Pathological features of tumor vasculature

### 2.1 Structural and functional alterations of tumor blood vessels

The normal vascular system is structurally organized, with endothelial cells arranged in a flattened monolayer and closely covered by pericytes, collectively maintaining vascular stability and barrier function. In contrast, tumor blood vessels exhibit interrelated abnormalities in structure, cellular composition, and function.

Structural abnormalities are the most apparent feature of tumor vasculature. Tumor blood vessels are tortuous and dilated, with disorganized branching and markedly increased permeability (3, 8). These abnormalities arise primarily from the persistent overproduction of pro-angiogenic factors, particularly VEGF, which drives continuous endothelial sprouting while preventing newly formed vessels from undergoing proper maturation, ultimately producing a chaotic vascular network (9–11).

Abnormalities in vascular cellular composition further impair vessel function. TECs are morphologically irregular and loosely organized, with disrupted intercellular junctions that create gaps in the vessel wall and increase plasma extravasation, leading to significantly elevated vascular permeability (12, 13). Meanwhile, reduced pericyte coverage deprives vessels of essential mechanical support, compromising vascular stability and exacerbating tissue hypoxia (14, 15). Together, these endothelial and pericyte abnormalities maintain tumor vessels in a chronically immature and structurally fragile state, impairing oxygen and nutrient delivery as well as barrier function (16) (**Figure 1A**).

**Figure 1:**
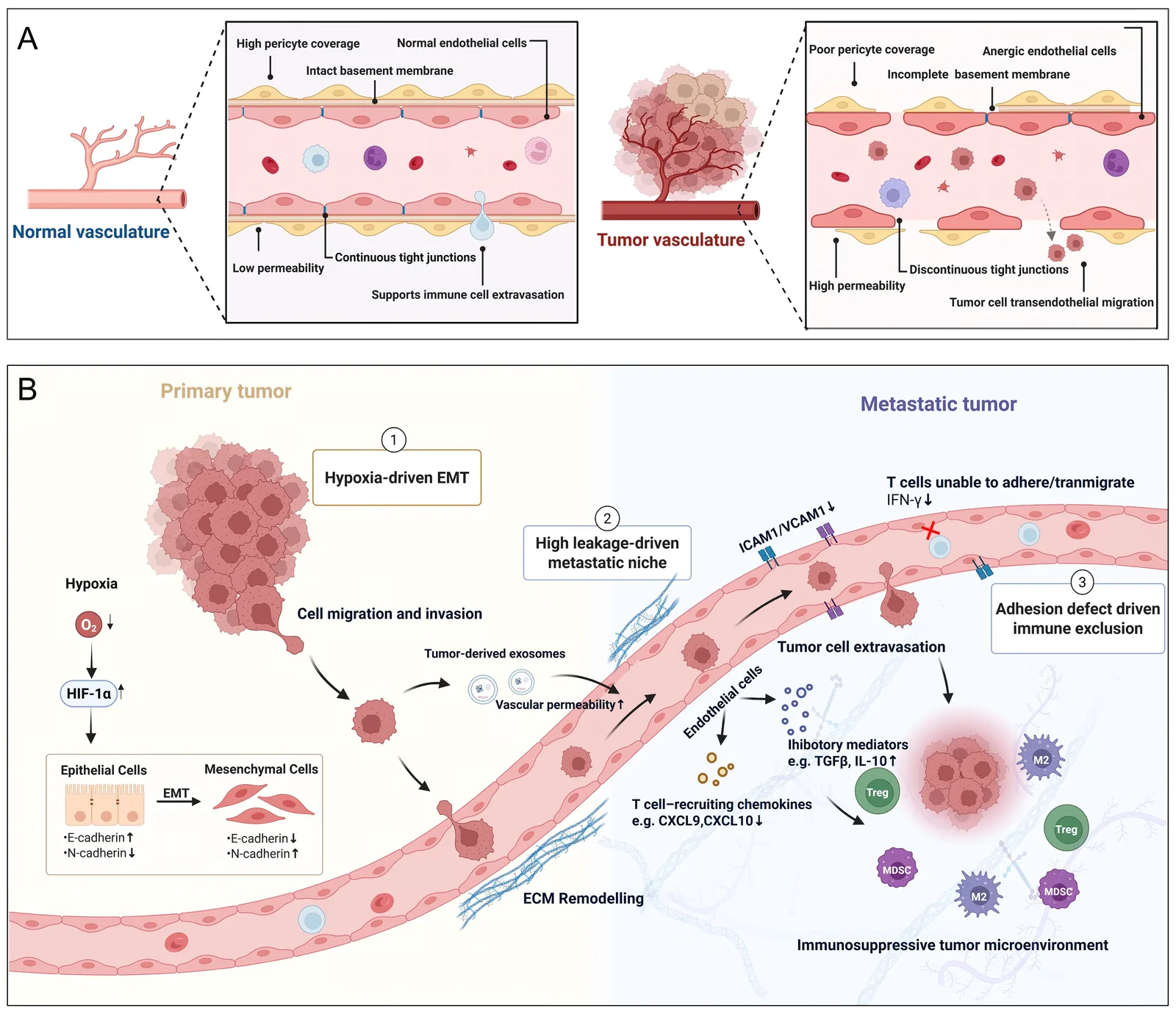
Pathological features of aberrant tumor vasculature and associated cascade responses. **(A)** Structural comparison between normal and aberrant tumor blood vessels. Normal vessels exhibit well-organized architecture with tightly aligned endothelial cells, intact pericyte coverage, and a continuous basement membrane. Tumor vessels display loosely arranged endothelial cells, reduced pericyte coverage, basement membrane disruption, and aberrant expression of junctional proteins. **(B)** Pathological cascade and metastatic progression driven by aberrant vasculature: ① Hypoxia in the primary tumor induces EMT, endowing tumor cells with migratory and invasive capabilities; ② Vascular hyperpermeability facilitates the establishment of a pre-metastatic niche through exosome-mediated signaling, ECM remodeling, and recruitment of immunosuppressive cells, thereby supporting tumor-cell extravasation and distant colonization; ③ Downregulation of endothelial adhesion molecules impairs T cell traversal across the vessel wall, resulting in an immunologically “cold” tumor with immune exclusion. These three processes are interconnected through the aberrant vascular system, collectively driving tumor metastatic progression.

These structural and cellular abnormalities culminate in vascular dysfunction. Tortuosity, leakage, and inadequate pericyte coverage impair intratumoral perfusion, causing severe hypoxia (17). Hypoxia subsequently induces the expression of pro-angiogenic factors such as VEGF, further aggravating vascular abnormalities and establishing a vicious cycle (18, 19). In addition, vascular leakage increases interstitial fluid pressure, which further impairs perfusion (20). Thus, structural abnormalities, cellular defects, and functional impairment interact to constitute the core pathological features of tumor vasculature.

### 2.2 Aberrant vasculature–driven pathological cascades

Tumor vascular abnormalities extend beyond structural and functional defects to reshape the tumor microenvironment and trigger interconnected malignant cascades.

#### 2.2.1 Hypoxia-driven epithelial-mesenchymal transition

Hypoxia is not merely a consequence of vascular abnormalities but a key driver of epithelial-mesenchymal transition (EMT) in tumor cells, enabling them to acquire migratory and invasive properties (21, 22). HIF-1α acts as a central molecular switch in this process by directly inducing multiple EMT-related transcription factors, thereby driving the loss of epithelial characteristics and acquisition of an aggressive mesenchymal phenotype (23). For example, in gastric cancer, hypoxia induces HIF-1α-dependent transcription of circPRELID2, which enhances PCBP1 O-GlcNAcylation to relieve the translational repression of ZEB2 mRNA, thereby promoting EMT and metastasis (24). Hypoxia can also establish self-reinforcing metabolic feedback. Under mild hypoxic conditions, activated HIF-1α transcriptionally suppresses cyclophilin D (CypD) expression, resulting in mitochondrial permeability transition pore (mPTP) closure, mitochondrial calcium accumulation, enhanced oxidative phosphorylation, and reactive oxygen species (ROS) production. ROS subsequently inhibit prolyl hydroxylases, further stabilizing HIF-1α under normoxia and forming a pseudohypoxic feedback loop that promotes EMT and melanoma metastasis (25). In addition to directly regulating EMT-related transcription factors, hypoxia can also induce EMT through intercellular signaling. In colorectal cancer, for instance, hypoxia stimulates cancer-associated fibroblasts to secrete CLEC11A, which activates the WNT/β-catenin pathway and promotes EMT (26). Thus, hypoxia-driven EMT not only enhances metastatic potential but also contributes substantially to resistance to diverse therapies (27).

#### 2.2.2 Vascular leakage and pre-metastatic niche formation

The pre-metastatic niche refers to a metastasis-promoting microenvironment that develops before tumor cell dissemination and is shaped by vascular leakage and endothelial cell reprogramming (28). Endothelial cells are key stromal components of the premetastatic niche, contributing not only to angiogenesis but also to immunosuppression, extracellular matrix remodeling, and inflammatory signaling (29). Tumor-derived extracellular vesicles (EVs) can disrupt endothelial tight junctions and adherens junctions, thereby increasing vascular permeability and facilitating tumor cell extravasation and distant colonization. In colorectal cancer, exosomal ADAM17 disrupts the membrane localization of vascular endothelial cadherin (VE-cadherin), thereby increasing vascular permeability, inducing pre-metastatic niche formation and promoting metastasis in nude mice (30). In colorectal cancer patients with liver metastasis, NEAT1-enriched EVs upregulate NKX2–3 in pericytes, which inhibits pericyte calcium influx through the PDE1C/cAMP/PKA signaling axis, consequently inducing vasodilation, increasing blood flow and vascular leakage, and promoting metastasis (31). Thus, vascular leakage-driven remodeling of the premetastatic niche not only facilitates the colonization of distant sites by circulating tumor cells but also contributes to metastatic progression and poor prognosis.

#### 2.2.3 Physical barrier to immune cell infiltration

Aberrant vasculature serves as a major physical barrier to immune cell entry into tumor tissues and contributes substantially to tumor immune evasion (32). Notably, vascular endothelial cells are not only essential components of this physical barrier but also active regulators of immune cell trafficking. Upon prolonged exposure to pro-angiogenic factors, TECs adopt an “anergic” state; these dysfunctional endothelial cells fail to upregulate adhesion molecules, including ICAM-1 and VCAM-1, thereby impairing immune cell adhesion and infiltration (3, 33). Beyond this passive obstruction, accumulating evidence indicates that TECs actively shape the immune landscape through angiocrine factors. For instance, downregulation of T cell–recruiting chemokines such as CXCL9 and CXCL10 in tumor endothelium limits the extravasation and intratumoral recruitment of CD8^+^ T cells (32, 34). Additionally, endothelial cells exacerbate local immunosuppression by secreting angiocrine factors that promote myeloid cell–mediated immunosuppression and suppress T cell activation (35). This vascular–immune crosstalk establishes a self-reinforcing feedback loop that amplifies the barrier effect: aberrant vasculature impedes T cell infiltration, while insufficient T cell activation reduces IFN-γ production, thereby limiting the restoration of vascular function and sustaining immunosuppression within the tumor microenvironment (36).

These three pathological cascades are not independent; rather, they are interconnected and mutually reinforcing. Hypoxia drives tumor progression and EMT while promoting vascular leakage; vascular leakage creates conditions for pre-metastatic niche formation; and the physical barrier formed by aberrant vasculature enables tumors to evade immune surveillance (**Figure 1B**). Together, these cascades form a central network underlying tumor invasion and metastasis and provide the pathophysiological basis for subsequent exploration of vascular normalization strategies.

## 3 Vascular normalization: from concept to molecular basis

### 3.1 Theoretical evolution: from “vessel destruction” to “vessel normalization”

In 1971, Folkman proposed that tumor growth is angiogenesis-dependent, laying the foundation for antiangiogenic therapy (37, 38). This vessel-destruction paradigm was subsequently reconsidered as evidence accumulated that tumor vessels are not merely therapeutic targets but also key regulators of the tumor microenvironment (39). In 2001, Jain introduced the concept of tumor vascular normalization, shifting the therapeutic focus from eliminating tumor vessels to restoring their structural and functional integrity (3, 4). Vascular normalization refers to the restoration of tumor vessels toward a phenotype that more closely resembles that of normal vessels (3, 40), characterized by increased pericyte coverage, an intact basement membrane, reduced vascular permeability, and improved perfusion. These changes alleviate tumor hypoxia and reduce interstitial fluid pressure, thereby enhancing drug delivery and immune cell infiltration (15, 41, 42). This theoretical shift established the basis for combining vascular normalization with immunotherapy.

### 3.2 Core biomarkers of vascular normalization

Accurate assessment of vascular normalization is essential for elucidating its molecular mechanisms, optimizing therapeutic regimens, and predicting treatment responses. Evaluation in preclinical and clinical studies primarily focuses on pericyte coverage, vascular permeability, and tissue oxygenation (11).

#### 3.2.1 Pericyte coverage

Pericyte coverage is a key indicator of vascular maturity and normalization. Pericytes are mural cells surrounding capillaries and postcapillary venules that interact with endothelial cells through direct contact and paracrine signaling to maintain vascular stability and barrier function (16, 43). In tumor vessels, pericyte coverage is markedly reduced and their attachment is often loose, contributing to vascular dysfunction. Due to the absence of a single specific marker, pericytes are typically identified using a combination of markers, including platelet-derived growth factor receptor β (PDGFR-β), chondroitin sulfate proteoglycan 4 (NG2), and α-smooth muscle actin (α-SMA) (15). During vascular normalization, pericytes are recruited to the endothelial surface and establish closer contacts, thereby increasing vascular maturity and improving perfusion (44). Bian et al. found that FOXF1 deficiency reduces pericyte coverage and exacerbates vascular leakage, whereas FOXF1 increases pericyte coverage through the FZD4/Wnt/β-catenin axis and inhibits lung cancer progression (45). Similarly, Lei et al. demonstrated that targeting the NAT10/XIST/YAP1 axis enhances pericyte coverage and preserves basement membrane integrity (44).

#### 3.2.2 Vascular permeability

Vascular permeability is a key indicator of tumor vascular function. In aberrant vessels, disrupted intercellular junctions and basement membrane cause plasma protein and fluid leakage, resulting in hyperpermeability, impaired blood flow, and facilitated tumor cell extravasation (46, 47). Vascular endothelial cadherin (VE-cadherin) (48) and claudin-5 (49, 50) are essential components of endothelial adherens and tight junctions, respectively, and are critical for vascular barrier integrity. In tumor vessels, both proteins are frequently downregulated or mislocalized and exhibit cross-regulatory interactions (51). During vascular normalization, their restored expression and localization at cell–cell contacts support barrier repair. Accordingly, vascular normalization has been associated with reduced permeability and restoration of VE-cadherin expression (29).

#### 3.2.3 Tissue oxygenation levels

Tissue oxygenation reflects the balance between oxygen supply and consumption and is a direct indicator of vascular perfusion and function. Vascular normalization alleviates tumor hypoxia by improving perfusion and oxygenation (2). Tumor oxygenation can be evaluated using histological, invasive, and non-invasive approaches. Pimonidazole staining, an exogenous bioreductive hypoxia marker, provides spatial information on hypoxic regions and is widely regarded as the “gold standard” for tumor hypoxia detection in preclinical and clinical studies (52, 53). Anti-angiogenic normalization reduces pimonidazole-positive areas, whereas endothelial-specific FOXF1 deficiency enlarges them and exacerbates hypoxia (45). Oxygen-electrode measurements and electron paramagnetic resonance (EPR) oximetry directly assess tissue oxygen tension but are limited by invasiveness or technical complexity. Non-invasive approaches include hypoxia-targeted positron emission tomography (PET), particularly ^18^F-fluoromisonidazole (^18^F-FMISO) PET, and functional magnetic resonance imaging (MRI) techniques such as blood oxygen level-dependent (BOLD), oxygen-enhanced (OE), and chemical exchange saturation transfer (CEST) MRI. The ^18^F-FMISO PET is among the best-established molecular hypoxia imaging methods, whereas BOLD and OE MRI provide radiation-free, repeatable alternatives (54–56).

Collectively, pericyte coverage, vascular permeability, and tissue oxygenation capture the structural and functional features of vascular normalization. Their combined assessment enables more accurate evaluation of normalization status and therapeutic efficacy, supporting both experimental research and clinical translation.

### 3.3 Molecular regulatory networks of vascular normalization

Tumor vascular normalization is an active process involving the coordinated modulation of multiple signaling pathways. Although these pathways are tightly regulated during physiological angiogenesis, they become persistently activated and interconnected within the tumor microenvironment, resulting in structural and functional vascular abnormalities. Targeting key nodes in these networks can therefore restore vascular integrity and promote vascular normalization (8, 57).

#### 3.3.1 Inhibition of excessive pro-angiogenic signaling

The VEGF family, comprising VEGF-A, VEGF-B, VEGF-C, VEGF-D, and placental growth factor (PlGF), is a central regulator of angiogenesis (58). Under physiological conditions, VEGF expression is tightly controlled. However, persistent VEGF overexpression in tumors drives excessive endothelial cell proliferation and migration, disrupts vessel maturation, and promotes the formation of a disorganized and highly permeable vascular network (59). Among these family members, VEGFA is the most extensively characterized and represents a major target of clinically used anti-angiogenic therapies. VEGFA signals primarily through VEGFR2, and anti-VEGF therapies such as bevacizumab inhibit this axis, promoting vascular normalization and alleviating vascular dysfunction and immunosuppression (60–62). Nevertheless, anti-VEGF therapy is just one of the clinically established anti-angiogenic approaches.

Beyond VEGF, other pro-angiogenic factors also contribute to tumor vascular dysfunction. Fibroblast growth factor (FGF) promotes endothelial cell survival and vascular branching through FGFR signaling and can act synergistically with VEGF (63). Platelet-derived growth factor (PDGF) is required for pericyte recruitment and vessel stabilization under physiological conditions, whereas its dysregulation in tumors can result in incomplete pericyte coverage, paradoxically exacerbating vascular instability and leakage (64). Aberrant activation of the angiopoietin-Tie2 pathway contributes to neovascularization and tumor progression in hepatocellular carcinoma (65), whereas IFN-γ can promote vascular normalization by suppressing the ANGPT2-Tie2 pathway in endothelial cells (66). Thus, targeting multiple dysregulated pro-angiogenic pathways may help restore vascular organization, reduce hyperpermeability, and improve vascular function, thereby promoting vascular normalization.

#### 3.3.2 Targeting hypoxia–HIF signaling to sustain vascular normalization

Hypoxia is a hallmark of the tumor microenvironment and a major driver of vascular abnormality. Hypoxia stabilizes HIF-1α, which transcriptionally activates multiple pro-angiogenic factors, including VEGF, PDGF, ANGPT2, and MMPs (67, 68). This creates a self-reinforcing loop between hypoxia and aberrant angiogenesis. Targeting the hypoxia-HIF axis therefore interrupts this cycle at the transcriptional level (69). Preclinical studies show that inhibiting HIF-1α or promoting its degradation reduces VEGF expression and normalizes tumor vasculature (70, 71). For instance, GSTZ1 deficiency in hepatocellular carcinoma inhibits PHD2 and stabilizes HIF-1α (72), whereas β-hydroxybutyrate suppresses the HIF-1α/VEGFA axis in colitis-associated colorectal cancer (73). Thus, targeting hypoxia–HIF signaling complements the direct inhibition of pro-angiogenic factors and helps sustain vascular normalization.

#### 3.3.3 Repairing extracellular matrix remodeling and mechanosensing abnormalities

The extracellular matrix (ECM) serves not only as structural scaffold but also as a dynamic signaling platform that regulates vascular behavior. Excessive deposition and remodeling of collagen and proteoglycans increase matrix stiffness and, together with abnormal mechanosensing, promote pathological angiogenesis (74). Overexpressed MMP-2 and MMP-9 degrade collagen, fibronectin, and laminin, compromising vascular basement membrane integrity (75). In parallel, tumor cells and cancer-associated fibroblasts promote ECM remodeling, which activates integrin-FAK/ERK signaling and enhances angiogenic activity (76). Integrin αvβ3, which is frequently overexpressed on TECs, further activates FAK and Src to promote endothelial migration and aberrant branching (77). Collagen-sensitive discoidin domain receptors (DDRs) can also activate RhoA/ROCK signaling in response to matrix stiffness, increasing cytoskeletal contractility and contributing to vascular dysfunction (78).

Mechanical cues are integrated primarily through the YAP/TAZ and RhoA/ROCK signaling pathways. Disturbed flow and low shear stress can activate YAP/TAZ through integrin- and GPCR-dependent mechanisms, thereby inducing pro-angiogenic genes such as CTGF and CYR61 (79, 80). In addition, the PIEZO1–YAP/TAZ-Notch axis contributes to tip/stalk cell specification during endothelial sprouting (81). Concurrently, YAP/TAZ can upregulate PD-L1 expression, thereby linking vascular abnormalities with immune evasion (82). Furthermore, the RhoA/ROCK pathway also responds to ECM stiffness and hemodynamic forces (83), wherein a stiff matrix activates RhoA through integrins, promoting myosin light chain phosphorylation, enhancing endothelial cell contraction, and leading to vascular lumen narrowing (84). The finding that ROCK inhibition reduces tumor vascular leakage further supports this pathway as a potential target for vascular normalization (85).

#### 3.3.4 Reversing metabolic reprogramming-driven tumor angiogenesis

Metabolic reprogramming is increasingly recognized as an important driver of tumor vascular dysfunction (86). Enhanced glycolysis causes lactate accumulation, which activates GPR81 on endothelial cells and macrophages and promotes VEGF expression, thereby promoting angiogenesis and vascular-associated immune evasion (87). In addition, lactate-associated L-2-hydroxyglutarate (L-2HG) production (88) and the accumulation of the oncometabolites succinate and fumarate (72, 89) can inhibit prolyl hydroxylases, stabilize HIF-1α, and increase VEGF expression. Succinate may also activate STAT3 and ERK signaling pathways through binding to the GPR91 receptor in a HIF-1α-independent manner, upregulating VEGF expression and driving angiogenesis (90, 91).

Metabolic reprogramming within endothelial cells also promotes pathological angiogenesis. Even under oxygen-rich conditions, endothelial cells primarily rely on glycolysis for energy production, with PFKFB3 serving as the key enzyme regulating this process (92). Within the tumor microenvironment, hypoxia and pro-angiogenic factors such as VEGF synergistically upregulate PFKFB3 expression in endothelial cells, leading to persistently elevated glycolytic flux (93). Enhanced fatty acid oxidation and glutamine metabolism may additionally support endothelial migration and proliferation (94, 95). Accordingly, PFKFB3, lactate transporters such as MCT4, and related metabolic pathways have emerged as potential therapeutic targets. PFKFB3 inhibitors, including PFK158, have shown promise in preclinical studies, while nanoparticle-based delivery may improve the vascular specificity of future metabolic interventions.

#### 3.3.5 Blocking the synergistic pro-angiogenic effects of immune cells and tumor cells

The reciprocal crosstalk between immune cells and tumor cells is a major driver of tumor vascular dysfunction (96). Hypoxia-induced HIF-1α activation promotes macrophage polarization toward a pro-angiogenic phenotype and enhances VEGF-mediated angiogenesis (97). Endothelial-derived ANGPT2 further recruits anti-angiogenesis-expressing tumor-associated macrophages (TAMs), amplifying pro-angiogenic signaling (98). Treg-derived TGF-β promotes VEGF expression (99) and PD-L1 induction (100), thereby impairing effector T-cell infiltration and function. TGF-β may also promote the conversion of tumor-infiltrating Th1 cells into Treg-like cells, further suppressing CD8^+^ T cell responses (101).

Tumor-derived exosomes provide an additional mechanism of immune–vascular crosstalk by transferring regulatory non-coding RNAs to immune cells (102). For example, glioblastoma stem cell-derived exosomal miR-374b-3p (103) and breast cancer cell-derived exosomal lncRNA HAGLROS (104) promote M2-like macrophage polarization, thereby facilitating angiogenesis and tumor progression. Collectively, these interactions shift immune cells toward pro-angiogenic and immunosuppressive states. Targeting tumor–immune crosstalk may therefore attenuate angiogenic signaling and support vascular normalization (**Figure 2**).

**Figure 2:**
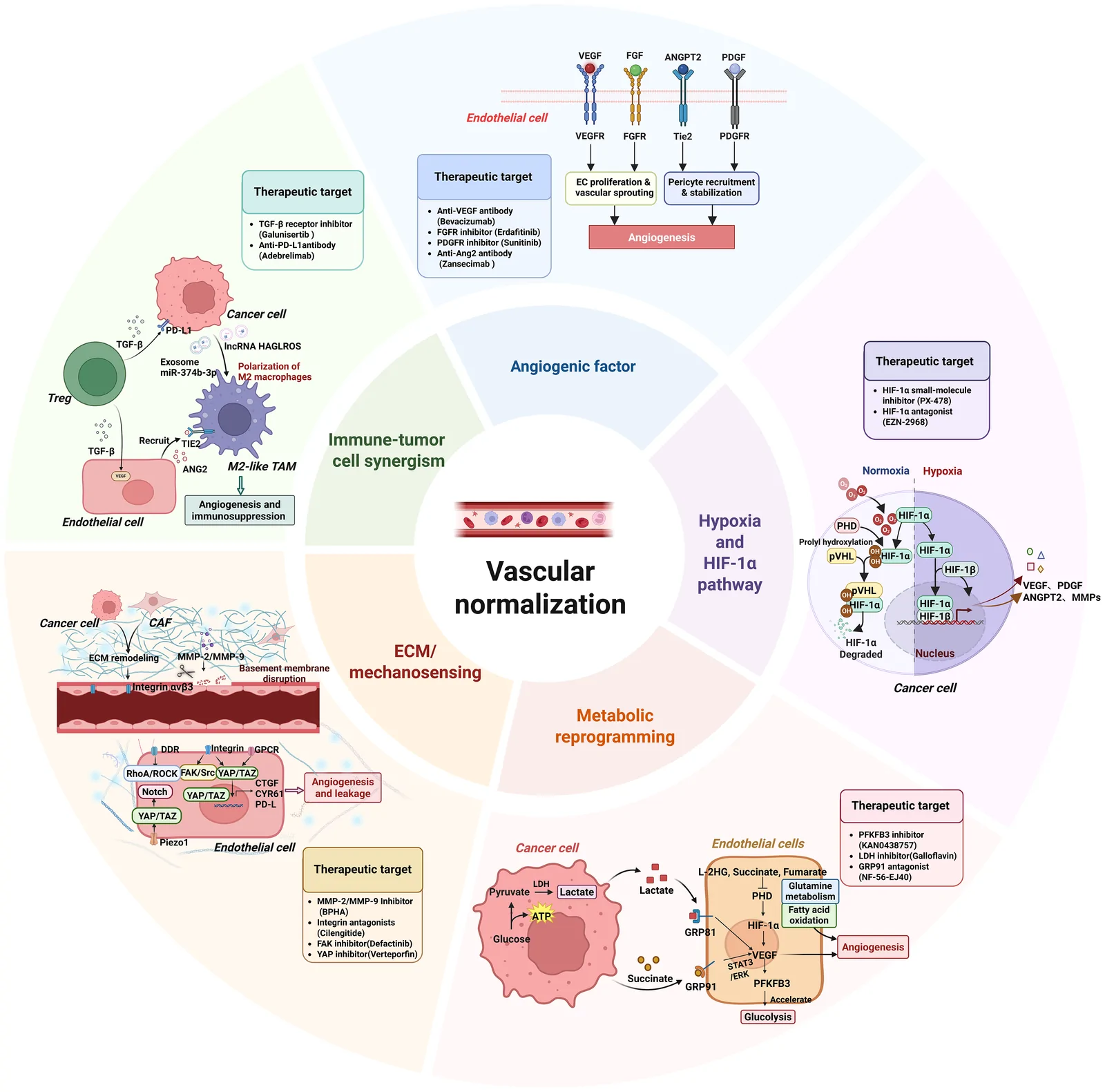
Multi-target regulatory network of vascular normalization. Five regulatory modules: angiogenic factors (e.g., VEGF and ANGPT2), HIF-1α signaling, ECM/mechanosensing, metabolic reprogramming, and immune cell interactions collectively contribute to tumor vascular abnormalities. Targeting key nodes within these modules reverses vascular structural disorganization and functional impairment, achieving vascular normalization. Normalized vessels further remodel the tumor immune microenvironment by alleviating immunosuppression and engaging an IFN-γ-mediated positive-feedback loop.

## 4 Vascular normalization remodels the tumor immune microenvironment

Vascular normalization improves tissue perfusion and drug delivery while remodeling the tumor immune microenvironment. By alleviating hypoxia, metabolic dysregulation, and the accumulation of immunosuppressive factors caused by aberrant vasculature, it can reduce immune evasion and restore antitumor immune activity. Conversely, activated immune cells may further support vascular repair, whereas normalized vessels facilitate immune-cell infiltration and function. This reciprocal interaction highlights the importance of vascular normalization in cancer immunotherapy and provides a rationale for developing combination treatment strategies.

### 4.1 Molecular mechanisms of vascular normalization in reversing immunosuppression

#### 4.1.1 VEGF signaling pathway and immunosuppression

The VEGF/VEGFR signaling pathway links aberrant angiogenesis to tumor immunosuppression (105, 106). In addition to promoting pathological vessel formation, sustained VEGF signaling induces endothelial anergy, characterized by reduced ICAM-1 and VCAM-1 expression, thereby impeding T cell transendothelial migration. Treatment with sunitinib or bevacizumab can restore ICAM-1 expression and enhance immune-cell infiltration, indicating that anti-VEGF therapy reverses endothelial anergy (107). Concurrently, VEGF also contributes to immunosuppression by impairing dendritic-cell maturation and antigen-presenting capacity (107), inducing T cell effector dysfunction (108), and promoting Treg proliferation (109). Anti-VEGF therapy can reduce PD-L1 expression in the tumor microenvironment (110) and attenuate the recruitment of Tregs and myeloid-derived suppressor cells (MDSCs), thereby relieving T cell suppression and improving effector T cell function (111). Therefore, by simultaneously restoring endothelial adhesion molecule expression and limiting immunosuppressive signaling and cell accumulation, VEGF/VEGFR blockade may reverse VEGF-mediated immune dysfunction and promote tumor immune microenvironment remodeling.

#### 4.1.2 Precise regulation of chemokines and immune cell recruitment

The chemokine network plays a critical role in vascular normalization-mediated immune microenvironment remodeling, promoting immune cell infiltration and functional restoration. In hepatocellular carcinoma, tumor-derived CCL16 interacts with ICAM-1 receptor on macrophages and activates the JAK2-STAT6 pathway, promoting IL-24 secretion and vascular normalization while alleviating immunosuppression (112). The CXCL12/CXCR4 chemokine axis also regulates tumor vascular function and immune-cell distribution. CXCL12 produced by fibroblasts or TECs promotes vascular permeability and abnormal vessel dilation (113, 114), recruits CXCR4^+^ bone marrow-derived cells to hypoxic angiogenic niches, and restricts the entry of CXCR4^+^ effector T cells into the tumor parenchyma (115). In hepatocellular carcinoma, the CXCR4 antagonist BPRCX807 reduces microvessel density and vascular abnormalities, decreases tumor-associated macrophage infiltration, and promotes cytotoxic T cell infiltration. Its combination with sorafenib or anti-PD-1 therapy further improves survival and suppresses distant metastasis in tumor-bearing mice (116). Similarly, endothelial PAK2 provides an additional link between vascular remodeling and immune recruitment. Endothelial-specific PAK2 deletion suppresses aberrant angiogenesis and increases CXCL10 production, thereby promoting the infiltration of dendritic cells and natural killer cells (117). Collectively, these findings identify chemokine axes as important targets for coordinating vascular normalization with immune activation.

#### 4.1.3 Bidirectional interplay between immune checkpoint blockade and vascular normalization

Immune checkpoint blockade (ICB) and vascular normalization interact bidirectionally, with IFN-γ serving as an important molecular link. ICB can promote vascular normalization by activating CD8^+^ T cells to produce IFN-γ, which suppresses ANGPT2/Tie2 signaling in endothelial cells (66). PD-1 blockade inhibits endothelial CXCL12-CXCR4 signaling, thereby reducing T cell exclusion and promoting a vascular phenotype that favors T cell infiltration (115). Similarly, metformin combined with PD-1 blockade enhances CD8^+^ T cell-derived IFN-γ signaling, thereby increasing endothelial VE-cadherin and VCAM-1 expression, promoting pericyte coverage, and improving vascular permeability and perfusion (118). Anti-CTLA-4 antibodies can activate CD4^+^ T cells in an Fc-dependent manner, inducing IFN-γ production and the formation of high endothelial venules that facilitate lymphocyte infiltration (119). Thus, ICB may directly contribute to vascular remodeling through effector T cell-derived IFN-γ.

Conversely, vascular normalization can enhance ICB efficacy by improving drug delivery and immune-cell infiltration. Restoration of endothelial junctions and basement-membrane integrity increases the penetration of anti-PD-L1 antibodies into tumor tissue (120). In cholangiocarcinoma, combining VEGF blockade with anti-CTLA-4 and anti-PD-L1 therapy promotes the conversion of Tregs toward an antitumor Th1-like phenotype through BAFF- and IL-12-dependent mechanisms, while enhancing proinflammatory B-cell responses (121). Moreover, nanoparticle-mediated delivery of MYC siRNA or miR-218 to endothelial cells normalizes tumor vessels, increases pericyte coverage and basement-membrane integrity, and enhances T cell infiltration and anti-PD-1 efficacy (122). Collectively, these findings support vascular normalization as a complementary strategy for improving ICB responses.

#### 4.1.4 Metabolic reprogramming and immune microenvironment remodeling

Metabolic reprogramming generates metabolites that act as signaling molecules and simultaneously drive vascular abnormalities and immunosuppression. Succinate, a cometabolite that accumulates in the tricarboxylic acid cycle, stabilizes HIF-1α by inhibiting PHD activity, thereby increasing VEGF expression and promoting tumor angiogenesis (72). It can also promote tumor-associated macrophages through HIF-1α-dependent signaling, thereby facilitating tumor metastasis (123). Lactate activates GPR81 on endothelial cells, promoting VEGF expression and angiogenesis (87). Concurrently, tumor-derived lactate can upregulate NUPR1 expression in macrophages through histone lactylation, thereby promoting M2 polarization and inducing CD8^+^ T cell exhaustion (124). Targeting metabolic reprogramming may therefore concurrently improve vascular and immune dysfunction. CPSF6 inhibition suppresses glycolysis and lactate production, reduces angiogenesis and PD-L1 expression, and increases the CD4^+^/CD8^+^ T cell ratio and granzyme B levels (125). Similarly, the bioresponsive nanoreactor ALMn depletes excess lactate, promotes vascular normalization, and shifts the tumor microenvironment toward an immune-activated state. Combined with anti-PD-L1 therapy, ALMn achieved an 83.7% tumor-inhibition rate in the reported model (126).

### 4.2 Feedback regulation of vascular normalization by immune cells

A complex bidirectional interplay exists between vascular normalization and immune microenvironment remodeling. Vascular normalization enhances the infiltration of immune cells, particularly CD8^+^ T cells, whereas activated immune cells can reciprocally regulate vascular structure and function through cytokines, proteases, and endothelial signaling.

The regulation of vascular normalization by immune cells is not unidirectional, but rather exhibits functionally opposing effects (**Table 1**). CD8^+^ T cells and Th1 cells promote vascular repair through IFN-γ secretion, whereas Tregs, MDSCs, and M2-like macrophages aggravate vascular dysfunction through pro-angiogenic mediators, including VEGF and MMP9. Thus, sustained vascular normalization may depend on the overall immune balance within the tumor microenvironment. Combination strategies should therefore not only increase T cell infiltration but also shift the functional balance away from immunosuppressive, pro-angiogenic immune populations to prolong vascular normalization.

**Table 1:** Feedback regulation of vascular normalization by immune cells.

Cell subset	Major effects on vascular function	Core molecular mechanisms	References
CD8^+^ T cells	Promote vascular normalization, suppress aberrant angiogenesis	Secrete IFN-γ, inhibit the ANGPT2-Tie2 axis, target and regulate endothelial cell function	(66, 127–129)
Th1 cells	Increase pericyte coverage, promote vascular normalization, alleviate tumor hypoxia	Secrete IFN-γ, regulate expression of angiogenesis-related genes (VEGF, ANGPT2) and adhesion molecules	(128, 130, 131)
M1-like macrophages	Promote vascular normalization, reduce leakage	Polarization switch from M2 to M1 phenotype, enhance secretion of pro-inflammatory cytokines such as IL-12 and TNF-α	(132–134)
M2-like macrophages	Promote tumor angiogenesis	Secrete multiple pro-angiogenic factors including VEGF and PDGF, as well as MMP-2/9, driving endothelial cell activation and basement membrane degradation to promote tumor angiogenesis	(134–140)
Treg cells	Promote tumor angiogenesis	Secrete VEGF; promote angiogenesis through hypoxia/CCL28 axis-mediated recruitment	(136, 141–143)
Natural killer (NK) cells	Promote vascular normalization, participate in vascular homeostasis maintenance	Secrete IFN-γ, regulate endothelial cell function; secrete pro-angiogenic factors or participate in vascular remodeling	(144–148)
Neutrophils	Suppress vascular normalization, disrupt vascular integrity, promote tumor angiogenesis	Form neutrophil extracellular traps (NETs) that disrupt vascular integrity and promote angiogenesis; secrete MMP-9 and VEGF to enhance vascular permeability and pathological angiogenesis	(149–154)
Myeloid-derived suppressor cells (MDSCs)	Suppress vascular normalization, promote tumor angiogenesis, increase vascular permeability	Secrete MMP-9 to degrade basement membrane, promote VEGF release and pathological angiogenesis	(155–158)

### 4.3 The tumor vascular system and formation of tertiary lymphoid structures

Vascular normalization improves perfusion and alleviates immunosuppression, facilitating T cell infiltration and providing a permissive vascular niche for tertiary lymphoid structure (TLS) formation (159, 160). TLS are ectopic lymphoid aggregates composed of B cells, T cells, dendritic cells, and high endothelial venules (HEVs) (161–163). As local hubs for antigen presentation and lymphocyte activation, mature TLS are associated with improved immunotherapy responses and prognosis and may serve as predictive biomarkers for immune checkpoint inhibitor therapy (164, 165).

The formation and function of TLS are closely linked to the tumor vascular system. HEVs mediate lymphocyte homing through glycosylated adhesion molecules, including peripheral node addressin (PNAd) (166, 167), and their abundance correlates with TLS density, immune-cell recruitment, and favorable clinical outcomes (168). HEVs provide an entry route for lymphocytes, whereas TLS support local antigen presentation and T cell activation (36). Spatial transcriptomics analyses further suggest that arterial endothelial cells within TLS can acquire HEV-like features through NOTCH downregulation, highlighting endothelial plasticity during TLS development (169).

Endothelial cells provide both structural support and signals that coordinate immune cell recruitment and organization (6). Under inflammatory stimulation, tumor and stromal cells release cytokines such as IL-1β, IL-6, and TNF-α, which activate NF-κB and STAT3 signaling and induce the expression of CXCL13, CCL19, and CCL21. These chemokines recruit lymphoid tissue inducer (LTi) cells, naïve lymphocytes, and dendritic cells to the tumor site, where they interact with endothelial cells to initiate TLS formation (170). Endothelial cGAS activation may further promote TLS formation through CCL5-mediated recruitment of CCR5^+^ CD8^+^ T cells. T cell-derived IFN-γ then enhances endothelial CCL5 secretion, forming a positive feedback loop that sustains T cell recruitment (171).

Consistent with the endothelial cell–driven mechanisms described above, anti-angiogenic therapy can promote vascular normalization and favor the development of tumor-associated HEVs and TLS (172). By improving perfusion, increasing pericyte coverage, and reducing hypoxia, normalized vessels support immune-cell infiltration; in turn, TLS-associated lymphocytes may reinforce vascular normalization through cytokine signaling (159, 160, 173). STING agonists, such as ADU-S100, may similarly promote vascular normalization and TLS formation by inducing TLS-associated factors, including lymphotoxin-α, IL-36, and type I interferons (160). The reciprocal relationship between vascular normalization and TLS formation supports the “vascular–TLS dual-biomarker system” proposed by Lei et al. Integrating regulation of the vascular normalization window with spatiotemporal assessment of vascular status and TLS formation may help optimize anti-angiogenic combination immunotherapy and address resistance to immune checkpoint blockade (159).

In summary, vascular normalization and TLS formation constitute a reciprocal regulatory network. Normalized vessels promote HEV development and lymphoid chemokine expression, facilitating TLS maturation; in turn, mature TLS sustain local antitumor immunity and may reinforce vascular normalization through cytokine-mediated signaling. This vascular–TLS interplay provides a rationale for anti-angiogenic immunotherapy combinations.

## 5 Molecular interfaces in tumor–endothelial–immune cell interactions

Tumor metastasis is a multistep, non-random process in which transendothelial migration of circulating tumor cells is a critical determinant of metastatic colonization. Rather than simply crossing a physical barrier, tumor cells engage in dynamic interactions with endothelial cells, immune cells, and the extracellular matrix (ECM) (174, 175). Like leukocytes, circulating tumor cells use selectins, integrins, and CD44 to mediate vascular rolling and firm adhesion. They further reprogram endothelial cells through soluble factors, exosomes, and surface receptor interactions, shifting the endothelium from a barrier toward a metastasis-supportive state. Endothelial cells also act as signaling hubs that regulate immune-cell recruitment and function through chemokines and immune checkpoint molecules. Based on these research advances, this section focuses on the complex molecular crosstalk among tumor cells, endothelial cells, and immune cells, and systematically elucidates the core mechanisms of multicellular interaction networks in tumor metastasis and immune regulation.

### 5.1 Molecular interface of tumor cell–endothelial cell interactions

Tumor cells intravasation and extravasation are critical steps in metastasis and depend on interactions between tumor cells and endothelial surface molecules (176). Beyond classical adhesion molecules, recent studies have identified additional ligand–receptor interactions that shape tumor–endothelial crosstalk.

#### 5.1.1 Classical adhesion molecules in tumor adhesion and transendothelial migration

The selectin family mediates the initial capture and rolling of tumor cells on the endothelium. Inflammatory cytokines and tumor-derived signals induce endothelial E-selectin and P-selectin, which bind sialylated Lewis X and A (sLeX/sLeA) ligands on tumor cells and facilitate subsequent firm adhesion (177). In bladder cancer, FUT7 overexpression increases sLeX modification of integrin α3 on small EVs, disrupting endothelial tight junctions and promoting vascular permeability and metastasis (178). In lung cancer, tumor-derived CCL2 activates endothelial cells, upregulates E-selectin, and promotes VE-cadherin dephosphorylation, thereby facilitating transendothelial migration (179). Moreover, bladder cancer-derived ITGA6^+^ EVs target lymphatic endothelial cells through ITGA6–CD151 interactions, inducing E-selectin-dependent lymphatic remodeling and lymph-node premetastatic niche formation (180). Endothelial cells also counteract selectin-mediated tumor adhesion through intrinsic regulatory mechanisms. ESDN suppresses E-selectin expression via STAT3 signaling; accordingly, ESDN deficiency increases tumor-cell adhesion and extravasation. Consistently, pharmacological inhibition of E-selectin with cimetidine attenuates these processes (181). Thus, selectin-mediated tumor-endothelial interactions are regulated by tumor-derived signals and endothelial-intrinsic feedback mechanisms.

ICAM-1 is another key adhesion molecule that promotes tumor-endothelial interactions in a cell context-dependent manner. In breast cancer, tumor-cell ICAM-1 supports circulating tumor cell cluster formation through homophilic ICAM-1 interactions and facilitates tumor-endothelial adhesion, transendothelial migration, and lung metastasis (182). In colorectal cancer, *Fusobacterium nucleatum* activates ALPK1/NF-κB signaling to upregulate tumor cell ICAM-1, thereby promoting endothelial adhesion, extravasation, and distant metastasis (183). Endothelial ICAM-1 can similarly impair vascular barrier function. Following incomplete radiofrequency ablation of hepatocellular carcinoma, heat stress upregulates ICAM-1 in tumor-associated endothelial cells. ICAM-1 then interacts with tumor cell ezrin, reduces VE-cadherin expression, and increases vascular permeability, facilitating tumor recurrence and metastasis (184).

CD44, the principal receptor for hyaluronic acid, also contributes to tumor cell adhesion and transendothelial migration. In lung cancer brain metastasis, CD44^+^ lung cancer stem cell-derived pericytes with high GPR124 expression activate Wnt7/β-catenin signaling, enhance transendothelial migration, and differentiate into tumorigenic cancer stem cells that form brain metastases (185). In adenoid cystic carcinoma, tumor-derived EVs containing circMYBL1 stabilize CEBPB and increase CD44 expression, promoting adhesion to pulmonary endothelial cells, tumor cell arrest, and lung metastasis (186). In addition, tumor-derived high-molecular-weight hyaluronic acid mediates endothelial adhesion through CD44, whereas CD44v isoforms can bind subendothelial ECM components to facilitate transendothelial migration and subsequent invasion (187).

#### 5.1.2 Functional analysis of emerging ligand-receptor pairs

The SLIT2-ROBO1 axis is an emerging mediator of tumor-endothelial interactions. Highly metastatic tumor cells release double-stranded RNA that activates endothelial TLR3, inducing SLIT2 expression. Endothelial-derived SLIT2 subsequently binds ROBO1 on tumor cells, promoting tumor cell chemotaxis toward endothelial cells and intravasation. Endothelial-specific SLIT2 deletion markedly reduces metastatic dissemination in breast and lung cancer models, supporting its role in transendothelial migration (188).

The ANGPT2-Tie2 axis regulates tumor vascular permeability and immune cell infiltration. Tumor-derived ANGPT2 binds to Tie2 receptors on endothelial cell surfaces, impairs peritumoral vascular integrity, and contributes to T cell exclusion and immunotherapy resistance. Accordingly, ANGPT2 blockade increases intratumoral CD8^+^ T cell infiltration and improves responses to immune checkpoint blockade (189).

Although these axes have been implicated in tumor-endothelial interactions, additional functional ligand–receptor pairs remain unidentified. Integrating single-cell sequencing with spatial transcriptomics may facilitate their systematic discovery and reveal potential targets for anti-metastatic therapy.

### 5.2 Endothelial cells as immune “gatekeepers”

TECs are not merely conduits for tumor-cell transendothelial migration but also act as critical “gatekeepers” of immune cell entry into the tumor parenchyma (190). Chronic exposure to pro-angiogenic factors induces endothelial anergy, characterized by reduced ICAM-1 and VCAM-1 expression, thereby impairing T cell adhesion and transendothelial migration (33, 107, 191). Endothelial cells also promote T cell apoptosis or dysfunction through FasL and PD-L1 expression (3).

Endothelial FasL forms an important immune barrier. FasL is selectively expressed by vascular endothelial cells in multiple human and mouse tumors, but is scarcely detectable in normal vessels. Tumor-derived VEGF-A, IL-10, and PGE_2_ synergistically induce endothelial FasL, enabling endothelial cells to selectively eliminate effector CD8^+^ T cells (192). Accordingly, integrin-targeting peptide-drug conjugates can reduce CD8^+^ T cell apoptosis by downregulating endothelial FasL expression, thereby inhibiting tumor growth and lung metastasis (193). Conversely, IL-12 combined with pulsed IL-2 promotes endothelial apoptosis through the IFN-γ–Fas/FasL pathway, producing anti-angiogenic and antitumor effects (194).

Endothelial PD-L1 and Galectin-1 also contribute to immunosuppression. Clinical analyses have revealed that high endothelial PD-L1 expression is associated with poor prognosis. In nasopharyngeal carcinoma, the tumor microenvironment activates the crosstalk between NF-κB and STAT3 signaling pathways in vascular endothelial cells, thereby upregulating PD-L1 expression and inducing CD8^+^ T cell functional exhaustion, promoting immune evasion (195). Tumor-secreted Galectin-1 reprograms TECs to express PD-L1 and Galectin-9, thereby restricting T cell infiltration; Galectin-1 blockade increases intratumoral T cell accumulation and enhances responses to radiotherapy and anti-PD-1 therapy (196). In hepatocellular carcinoma, Galectin-1 from TREM2^+^ tumor-associated macrophages further promote endothelial PD-L1 expression and suppresses CD8^+^ T cell recruitment (197).

Single-cell and spatial transcriptomic studies indicate that endothelial gatekeeping is mediated by functionally distinct TEC subsets rather than uniformly across the endothelium. In pancreatic ductal adenocarcinoma, RASIP1^+^ TECs express high ICAM1 and colocalize with CD8^+^/granzyme B^+^ T cells, suggesting a role in facilitating T cell entry (198). Conversely, deep-region endothelial cells in stem-like gastric cancer activate the VISFATIN-NAMPT-ITGA5 axis and are enriched in ICB non-responders (199). In hepatocellular carcinoma, S100A16^+^ TECs are highly enriched in tumor tissues and drive HCC progression through coordinated promotion of pathological angiogenesis and immunosuppression (200). Pan-cancer analyses further identify CXCR4^+^ tip cells and SELE^+^ venous endothelial cells as angiogenic and proinflammatory TEC phenotypes, respectively; tumors show increased CXCR4^+^ tip cells but reduced SELE^+^ venous cells compared with adjacent tissues, a shift associated with treatment responses to anti-angiogenic therapy and immunotherapy (7). Collectively, TECs regulate T cell infiltration through diverse mechanisms and specialized subsets. Selectively targeting immunosuppressive TECs while preserving immune-permissive populations may improve immunotherapy efficacy.

### 5.3 Pericytes in vascular-immune interactions

Pericytes, traditionally viewed as structural supporters of capillaries, are now recognized as paracrine regulators of immune cell recruitment, polarization, and function in the tumor microenvironment (43, 201, 202). Single-cell multi-omics and spatial transcriptomics analyses have identified a PRRX1-driven GPR116^+^ pericyte subset enriched in metastatic tumors and associated with poor survival and resistance to immunotherapy. These pericytes promote tumor progression by secreting EGFL6, which activates NF-κB signaling and induce EMT, while the ALOXE3–12-HETE–GPR31 axis promotes CD8^+^ T cell exhaustion, Treg expansion, and PD-L1 expression. Targeting GPR116 or integrin β1 suppresses metastasis and enhances anti-PD-1 efficacy (203).

Pericyte phenotypic switching also shapes the immune microenvironment. Synthetic pericytes secrete inflammatory factors, including IL-6, to promote tumor-associated macrophage infiltration and sustain immunosuppression. In contrast, differentiated or contractile pericytes reduce inflammation, support vascular normalization, alleviate hypoxia, and enhance responses to adoptive T cell therapy (201, 204). Low-dose inhibition of MEK, AKT, or Notch signaling, as well as microtubule-binding drugs such as eribulin and combretastatin A4, can promote this transition through Rho/ROCK-related signaling (201, 204) (**Figure 3**).

**Figure 3:**
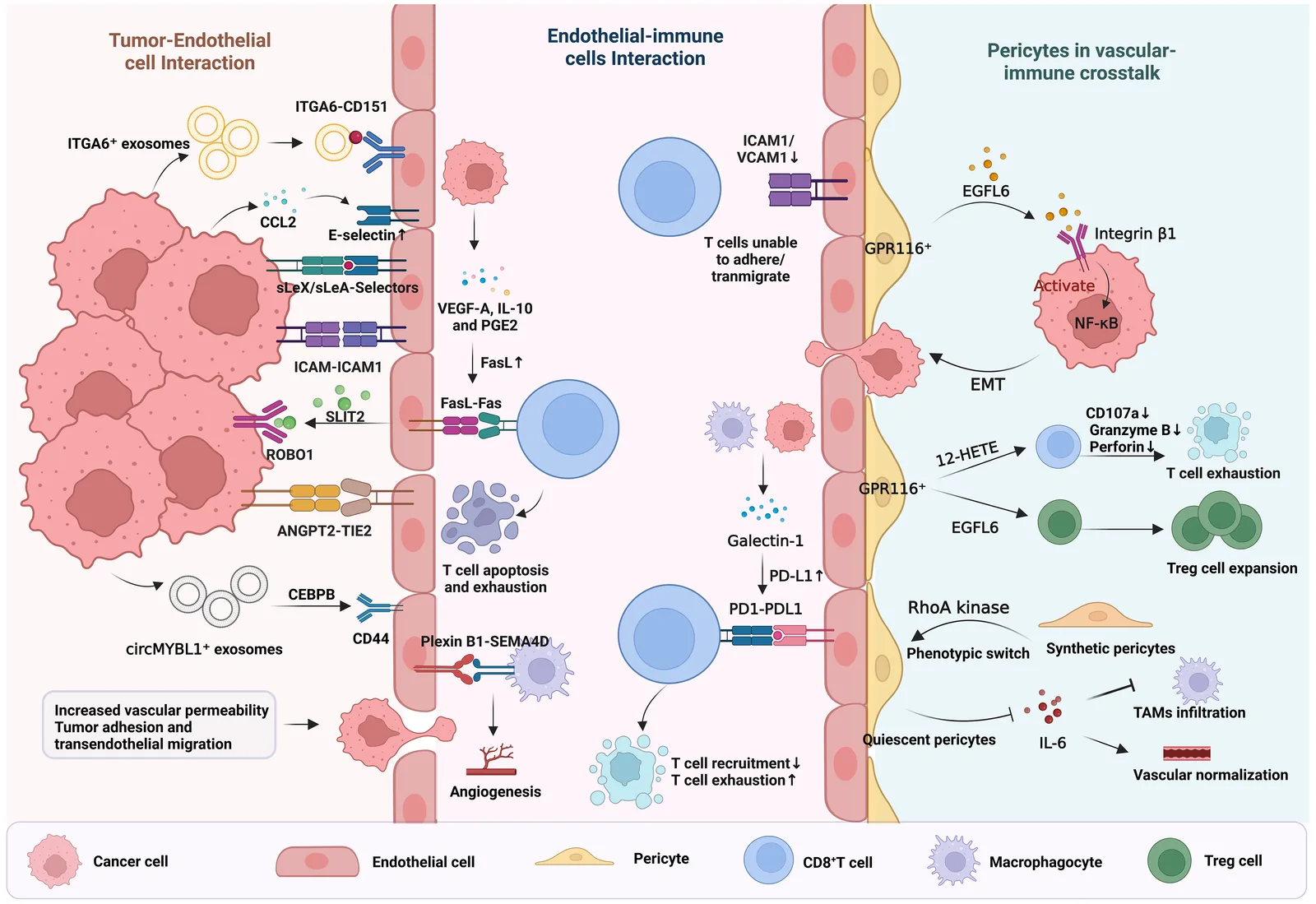
Molecular interfaces in tumor–endothelial–immune cell interactions. Tumor cells directly interact with endothelial cells through adhesion molecules, including selectins and ICAM-1, and ligand–receptor pairs such as SLIT2–ROBO1 and ANGPT2–Tie2. They also modulate endothelial function through exosome-mediated paracrine signaling (Left). Endothelial cells act as immune gatekeepers by inducing T cell apoptosis or exhaustion via FasL and PD-L1, or by facilitating T cell transmigration through ICAM-1 and VCAM-1. Tumor-associated macrophage-derived SEMA4D binds to endothelial Plexin B1 to induce angiogenesis (Middle). Pericytes construct immunosuppressive barriers through the GPR116^+^ subset and the EGFL6/ALOXE3–12-HETE secretory axis, while phenotypic switching between synthetic and differentiated states further regulates the immune microenvironment (Right).

In summary, tumor cells, endothelial cells, and pericytes form an interconnected network that regulates transendothelial migration, vascular function, and local immunosuppression. Defining this network may support the development of anti-metastatic and immunotherapy-based combination strategies.

## 6 Conclusions and perspectives

Tumor growth and metastasis depend heavily on aberrant tumor vasculature. This review summarizes the pathological features and underlying mechanisms of vascular abnormalities, and examines how vascular normalization remodels the tumor immune microenvironment through interactions among tumor cells, endothelial cells, pericytes, and immune cells. By restoring vascular structure and function, vascular normalization can alleviate hypoxia and immunosuppression, enhance T cell infiltration, and promote reciprocal reinforcement between vascular repair and immune activation. Its bidirectional relationship with tertiary lymphoid structures (TLS), together with multicellular vascular–immune crosstalk, provides a conceptual basis for optimizing combination therapies (**Figure 4**).

**Figure 4:**
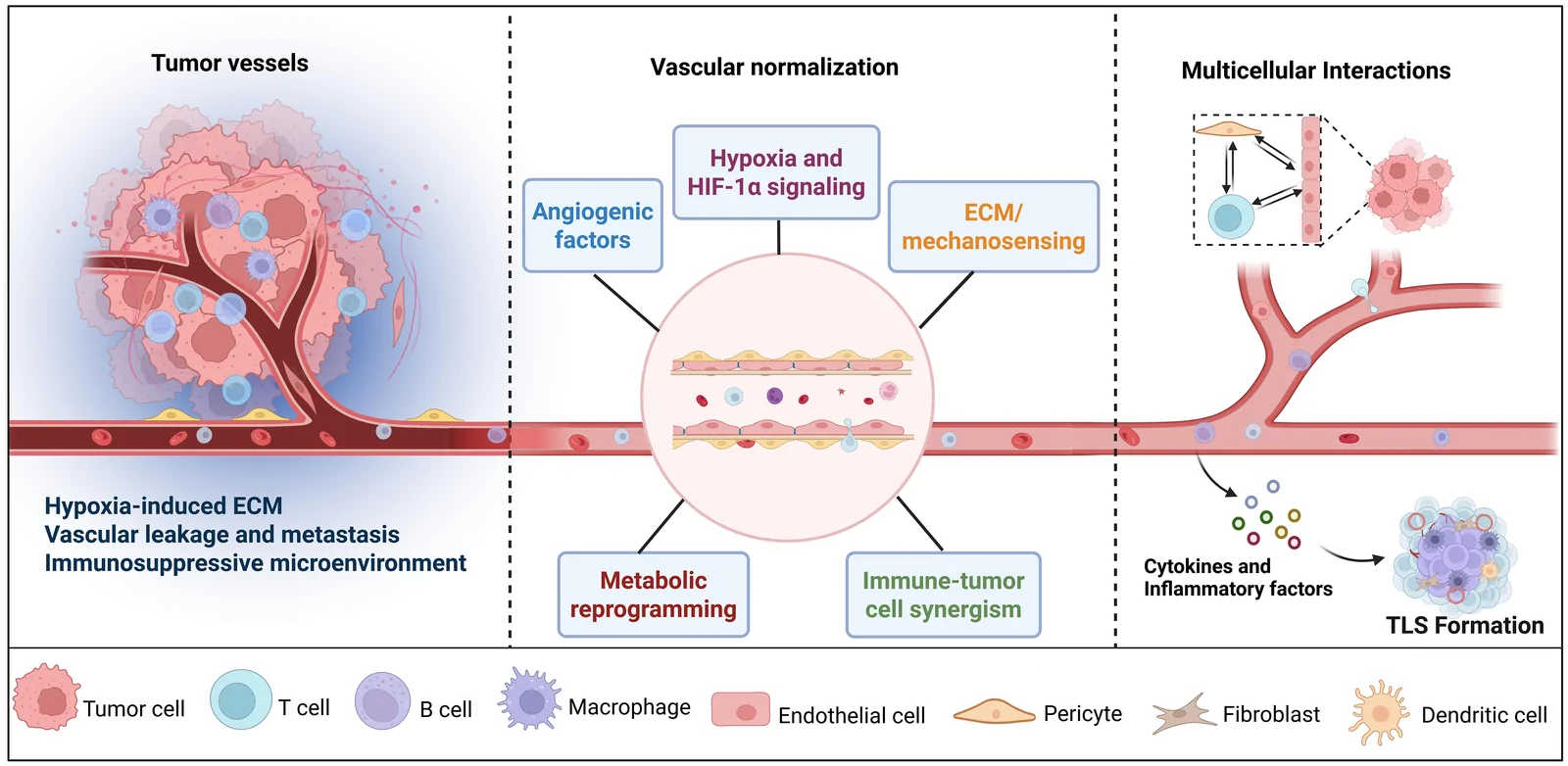
Summary framework of vascular normalization, multicellular interactions, and TLS formation. Aberrant tumor vessels drive hypoxia, vascular leakage, and immunosuppression (left). Vascular normalization is achieved by targeting five regulatory modules (angiogenic factors, hypoxia/HIF-1α, ECM/mechanosensing, metabolic reprogramming, and immune–tumor synergy), restoring vessel structure and perfusion (middle). Normalized vessels facilitate immune-cell recruitment, multicellular vascular-immune interactions, and cytokine signaling, thereby supporting TLS formation (right).

Despite its therapeutic potential, the clinical translation of vascular normalization remains challenging. Defining the normalization window and developing reliable biomarkers for longitudinally monitoring vascular status and treatment response are critical. Tumor heterogeneity, functionally distinct TEC subsets, the transient nature of the normalization window, and adaptive resistance to prolonged anti-angiogenic therapy may limit treatment efficacy. In addition, the complex signaling interactions among pericytes, endothelial cells, and immune cells remain incompletely understood, and effective strategies for targeting these cellular interfaces are still lacking.

Future research should move beyond static biomarkers toward dynamic, spatially resolved analyses of vascular-immune networks. Integrating a vascular–TLS dual-biomarker framework with spatial transcriptomics and longitudinal sampling may clarify the spatiotemporal relationship between vascular status and TLS formation, identify optimal treatment windows and immune-permissive regions, and guide personalized combination therapy. Therapeutic development should also focus on cellular interfaces rather than entire cell populations. Low-dose strategies that induce pericyte phenotypic switching, together with multispecific biologics or engineered peptides targeting key tumor-endothelial-pericyte interactions, may sustain vascular remodeling and limit metastasis while preserving lymphocyte trafficking. Proximity labeling and single-cell multi-omics could further identify regulatory nodes in tumor-endothelial crosstalk and resolve TEC heterogeneity, enabling selective targeting of immunosuppressive TEC subsets while preserving immune-permissive populations.

Overall, vascular normalization provides a framework for coordinated vascular and immune remodeling. Defining multicellular interaction networks and developing dynamic biomarkers and interface-specific therapies will be essential for overcoming immunotherapy resistance and advancing precision cancer treatment.
